# A noninvasive method for assessing optimal cerebral perfusion pressure

**DOI:** 10.1186/s13054-026-05926-w

**Published:** 2026-03-11

**Authors:** Sérgio Brasil, Gustavo Frigieri, Fabio Silvio Taccone, Diogo Dantas, Carlos Nassif, Marek Czosnyka, Pedro Kurtz, Salomon Soriano Ordinola Rojas, Wellingson Silva Paiva, Luiz Marcelo Sá Malbouisson

**Affiliations:** 1 Division of Neurosurgery, Department of Neurology, University of São Paulo Medical School, Av. Dr. Eneas de Carvalho Aguiar 255, São Paulo, Brazil; 2 Medical Investigation Laboratory 62, University of São Paulo Medical School, São Paulo, Brazil; 3 Brain4care, São Paulo, Brazil; 4 Department of Intensive Care, Hospital Erasme, Universitè Libre de Bruxelles, Brussels, Belgium; 5 Department of Anesthesiology, University of São Paulo Medical School, São Paulo, Brazil; 6 Intensive Care Unit Hospital 9 de Julho, São Paulo, Brazil; 7 Department of Clinical Neurosciences, Division of Neurosurgery, University of Cambridge, Cambridge, UK; 8 Intensive Care Department, Instituto Estadual do Cérebro, Rio de Janeiro, Brazil; 9 Intensive Care Department coordinator, Hospital Beneficência Portuguesa de São Paulo, São Paulo, Brazil

**Keywords:** Acute brain injury, Intracranial pressure, Cerebrovascular autoregulation, Nnoninvasive neuromonitoring

## Abstract

****Introduction**:**

After acute brain injuries, optimizing cerebral perfusion pressure (CPP) is critical for preventing secondary brain insults, yet current fixed CPP targets may not be ideal for all patients due to individual variability in cerebrovascular autoregulation (CA). The concept of"optimal CPP" (CPPopt) or "optimal mean arterial pressure" (MAPopt) identifies the specific pressure range where CA is most effective. The Pressure-Reactivity Index (PRx), derived from invasive intracranial pressure (ICP) and arterial blood pressure (ABP) monitoring, is a well-established means for assessing CA and MAPopt. This study aimed to investigate the ability of noninvasive surrogate ICP waveforms, specifically the P2/P1 ratio acquired using a cranial deformation sensor (B4C), to determine MAPopt in patients with acute brain injury, and to compare its efficacy with the established invasive PRx method.

**Methods:**

This paper provides a retrospective analysis of data from a multicenter prospective observational study of intensive care patients with severe brain injuries requiring invasive ICP monitoring. Continuous invasive ABP and ICP data were collected alongside noninvasive ICP waveforms using the B4C sensor. MAPopt was determined for each monitoring session using two methods: (1) the nadir of a polynomial regression curve fitted to PRx values (correlation between ICP and ABP) stratified by 1 mmHg MAP intervals, and (2) the nadir of a polynomial regression curve fitted to the noninvasive P2/P1 ratio, also stratified by MAP intervals. Repeated measures correlation was used to analyze their correspondence and P2/P1 ratio ranges within MAPopt limits, whereas Bland-Altman analysis for the methods agreement.

****Results**:**

A total of 114 patients were included in the study, 68% severe traumatic brain injury and 15% spontaneous subarachnoid hemorrhage. Analysis of optimal MAP for each session revealed a strong linear relationship between the MAPopt derived from the invasive PRx and the noninvasive P2/P1 ratio (r = 0.905, p<0.0001). Bland-Altman analysis demonstrated good agreement between the two methods, with a mean difference of+2.00 mmHg and 95% limits of agreement ranging from −9.87 mmHg to +13.86 mmHg.

****Conclusion**:**

The noninvasive P2/P1 ratio, serving as a marker of intracranial compliance, may effectively and accurately infer the optimal systemic mean arterial pressure. A noninvasive neuromonitoring tool may enable personalized, bedside-guided therapeutic management, significantly widening the assessment of cerebrovascular autoregulation in critical care settings.

**Supplementary Information:**

The online version contains supplementary material available at 10.1186/s13054-026-05926-w.

## Introduction

Acute brain injury (ABI), encompassing conditions such as traumatic brain injury (TBI), subarachnoid hemorrhage (SAH), and ischemic stroke, represents a leading cause of morbidity and mortality worldwide [1, 2]. Patients in neurocritical care settings are particularly vulnerable to secondary brain insults, which can significantly worsen neurological outcomes. A cornerstone of neurocritical care management is the optimization of cerebral perfusion pressure (CPP) and mean arterial pressure (MAP) to ensure adequate cerebral blood flow (CBF) and oxygen delivery to the brain [3]. However, maintaining CBF is complicated by the frequently impaired state of cerebrovascular autoregulation (CA) in injured brains [4].

Cerebral autoregulation is a complex physiological mechanism that allows the cerebral vasculature to maintain a relatively constant CBF over a wide range of MAP fluctuations [5]. Traditional neurocritical care guidelines often recommend fixed CPP thresholds, typically ranging from 60 to 70 mmHg [6]. However, growing evidence suggests that these generalized targets may not be optimal for all patients, as the ideal CPP can vary significantly between individuals and even within the same patient over time, depending on the dynamic state of their CA [7]. The concept of “optimal CPP” has emerged, defined as the CPP range where CA is most effective, thereby possibly maximizing cerebral oxygenation and minimizing secondary injury [8].

The Pressure-Reactivity Index (PRx) is a real-time monitoring tool for assessing the integrity of CA and the means of optimal CPP identification [5]. PRx is calculated as a moving correlation coefficient between mean intracranial pressure (ICP) values and ABP [9]. Physiologically, when CA is intact, an increase in ABP leads to vasoconstriction, preventing a significant rise in ICP, resulting in a negative or near-zero PRx. Conversely, when CA is impaired, changes in ABP are passively transmitted to the cerebral vasculature, causing concomitant changes in ICP. This leads to a positive PRx, indicating exhausted autoregulatory capacity. However, the primary drawback of PRx is its dependence on invasive ICP monitoring (typically requiring an intraparenchymal or ventricular catheter) and continuous arterial pressure monitoring. Such approach has limited applicability, being restricted to critically ill patients, especially in TBI. Therefore, when ICP monitoring is unavailable or contraindicated, PRx and CPPopt identification are precluded.

It has been demonstrated that ICP waveform can reflect CA state, changing its morphology following ICP variations but also cerebral hemodynamics [10–12]. This study hypothesized that ICP waveforms may help assess optimal MAP. Therefore it was examined whether noninvasive ICP waveforms from a cranial deformation sensor can indicate optimal MAP by comparing them to invasive PRx measures. This approach could represent a step forward in advanced neuromonitoring and a remarkable strategy in individualized care, widening CA assessment at the bedside.

## Methods

### Study design

This study retrospectively analyzes a prospective multicenter database which was established to assess the association between qualitative and quantitative invasive ICP monitoring data and the novel skull extensometer (B4C) in patients with ABI. The current method enabled the evaluation of PRx and optimal MAP by invasively recording arterial blood pressure waveforms and values from radial arteries. This study aims to establish a proof-of-concept for a PRx surrogate parameter, without drawing clinical or prognostic correlations. Future research with a larger, longer-term cohort is needed for such analysis.

The observational study took place in the intensive care units of Hospital das Clínicas (São Paulo University, Brazil) and was approved by the local Ethics Committee (registered at clinicaltrials.gov NCT03144219). Hospital João XXIII, under the reference number 6,150,621, CAAE: 39348920.1.1001.0068; Federal University of São Paulo, under the reference number 3.129.120, CAAE: 03843118.0.0000.5505; Hospital Estadual de Emergência e Trauma Senador Humberto Lucena, under the reference number 5.078.425, CAAE: 39348920.1.2001.5186; University of Porto, under the reference number 106–17; Stanford University, under the reference number 46,100. Consent was collected from legal representatives or next of kin before including patients. The study followed the Standard for Reporting of Diagnostic Accuracy studies (STARD) guidelines (Supplemental).

### Population

Only patients with severe TBI, SAH, intracranial hematomas or ischemic stroke who received an invasive ICP monitor, as per Brain Trauma Foundation guidelines and neurosurgical/neurointensivist recommendations, were included [6, 13]. The inclusion criteria for this study was invasive ICP and an invasive ABP line for continuous beat-by-beat monitoring. Study investigators did not interfere with medical management. Consecutive patients were enrolled within 48 h of hospital admission, with all recordings conducted during the initial week of hospitalization while ICP monitoring was ongoing. The number of recording sessions varied per patient, ranging from one to three, as the primary objective was to correlate invasive and noninvasive ICP waveforms. Data collection commenced after patients had received all required urgent interventions, including sedation, intubation, ICP monitor placement, or intracranial mass evacuation when required. Continuous monitoring of invasive ABP, electrocardiogram, temperature, and oxygen saturation is standard practice. In these institutions, in cases of non-traumatic neurosurgical emergencies deemed at risk for brain herniation by the clinical team, ICP monitoring is implemented.

During the study, patients were deeply sedated (Richmond Agitation Sedation Scale ≤−4) along the recordings. Exclusion criteria included prior decompressive craniectomy or persistent fixed mydriatic or mid-sized pupils for over two hours post-stabilization. Data collected: age, sex, admission diagnosis, admission Glasgow coma score, computed tomography scores, ABP, ICP, PRx, and P2/P1 ratio.

### Neuromonitoring and data collection model

Spontaneous ABP fluctuation was recorded invasively with a radial artery catheter. The pressure transducer was leveled and zeroed to the intersection of the anterior axillary line and the fifth intercostal space. No ABP manipulations were done for study purposes; variations occurred spontaneously or as part of routine patient management by local teams. ICP values were recorded with the Neurovent system (Neurovent-P when parenchymal and Neurovent when external ventricular drain) using an optic-fiber transducer (Raumedic, Munchberg, Germany).

**Fig. 1:**
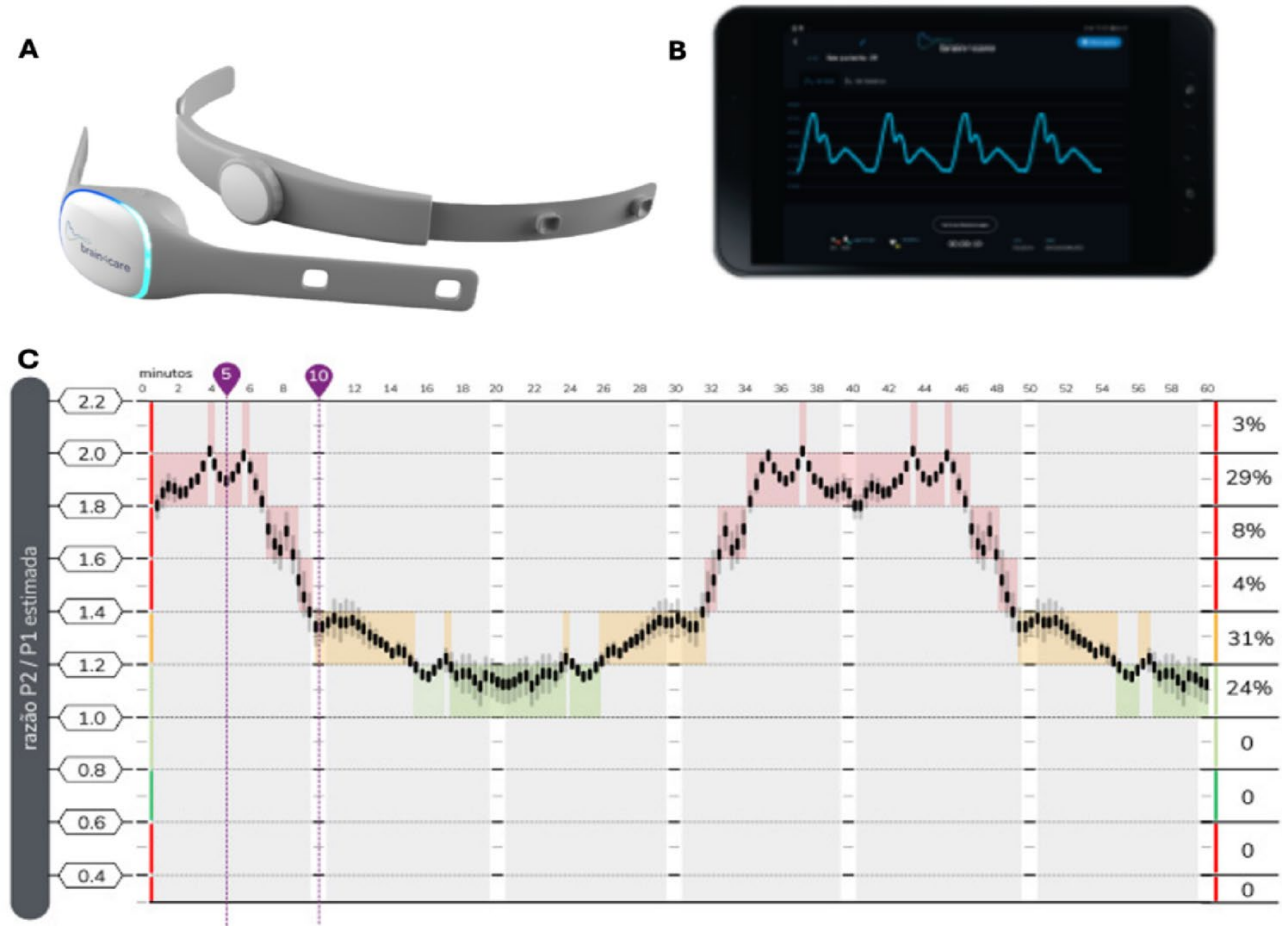
The B4C system. **A** - The noninvasive wireless sensor is placed around the patient’s head. **B** - Real-time waveforms are displayed within the app. **C** - The live report shows P2/P1 ratio trends during a one-hour monitoring session

Simultaneously, noninvasive ICP waveform (nICPW) was measured with the FDA-cleared B4C sensor (brain4care, São Carlos, Brazil; K201989). Previous studies described its features and principle of operation in detail [14, 15]. The B4C system has a highly sensitive surface to be placed on the skin over the skull to detect tiny cranial expansions caused by changes in intracranial pressure with each heartbeat (Fig. 1). B4C provides the P2/P1 ratio for clinicians at the bedside, which is a marker of intracranial compliance [16–19]. This is given from dividing the second peak (P2) amplitude by the first peak (P1) amplitude found in ICP waveform. Previous studies demonstrated the correlation of this ratio/index with ICP [12, 20]. Multiple clinical studies consistently show these waveforms correlate with their invasive predicate [11, 15, 20–23]. The B4C device was positioned in the frontotemporal region ipsilateral to the site of ICP probe implantation.

Data acquisition involved several recording sessions, each with an approximate duration of 10 min and a sampling frequency of 250 Hz. The investigators were trained to closely monitor each session to ensure the B4C sensor remained in place and signal quality was not affected [24]. The collected waveforms (B4C, ICP, and ABP) were displayed in real time on both the multiparametric monitor and the computer used for data recording. Since all monitoring sessions in the study were closely supervised by the research team, there were no instances of missing data (signal lost).

### Data analysis

Artifacts, when identified by the analytics team, were removed after visual inspection. The coefficients between the input (ABP) and the outputs (ICP for PRx and P2/P1 ratio for B4C) were computed with a 5-minute moving window and 10-second step. Data were grouped into 1 mmHg MAP intervals. The optimal MAP was determined as the lowest point of a polynomial regression curve fitted to these intervals, shown as boxplots.

### Statistical analysis

Demographic and clinical characteristics of the patients were described using mean and standard deviation or median and interquartile range for continuous variables, and absolute and relative frequencies for categorical variables. Mean P2/P1 ratio and PRx values were calculated to show their distributions. PRx has clinically established cutoffs (e.g., PRx > 0.3 or 0.25) for distinguishing between intact and impaired cerebral autoregulation [25, 26]. Correlation coefficients were then calculated to measure the relationship between them. To evaluate the within-subject correlation between optimal MAP PRx and optimal MAP P2/P1 ratio, while accounting for the non-independence of observations, we employed repeated measures correlation (rmcorr) [27]. Bland-Altman analysis evaluated agreement between values of optimal MAP calculated using P2/P1 and PRx, including systematic bias and 95% limits of agreement. All statistical analyses were performed in Python 3.12 using the Pingouin statistical library. Results were presented as correlation coefficient r and 95% confidence intervals (CI). Statistical significance was set at *p* < 0.05.

## Results

A total of 376 monitoring sessions from 178 patients were collected. Nearly all patients underwent two sessions, each lasting approximately 30 min. Since session lengths ranged from 10 to 40 min, only sessions longer than 15 min were included to allow more 10-second windows and improve assessment precision, yielding a final sample of 114 patients and 268 sessions. Data loss was avoided in this instance as a result of the study’s methodology. Severe TBI represented the most prevalent condition within the sample. Demographics and mean values for the parameters of interest are shown in Table 1.

**Table 1:** Characteristics of the included population, presented as percentages, mean and standard deviation (SD)

114 patients		
Age (years)	45.7 mean	17.9 SD
Sex	67 Male	58%
Admission GCS	5.4 mean	2.2 SD
ICP (mmHg)	13.1 mean	10 SD
ABP (mmHg)	91.5 mean	13.5 SD
P2/P1 ratio	1.17 mean	0.3 SD
TBI	78	68%
Admission CT	Marshall 4–56	71%
	Marshall 3–22	28%
SAH	18	15%
Admission CT	Modif. Fisher 4–18	100%
ICH	12	10%
IS	6	7%
Craniotomy	74	64%
EVD ICP	89	78%
Parenchymal ICP	25	21%

ABP: arterial blood pressure, CT: computed tomography, EVD: external ventricular drain ICH: intraparenchymal hematoma, ICP: intracranial pressure, GCS: Glasgow Coma Scale, IS: ischemic stroke, TBI: traumatic brain injury

**Fig. 2:**
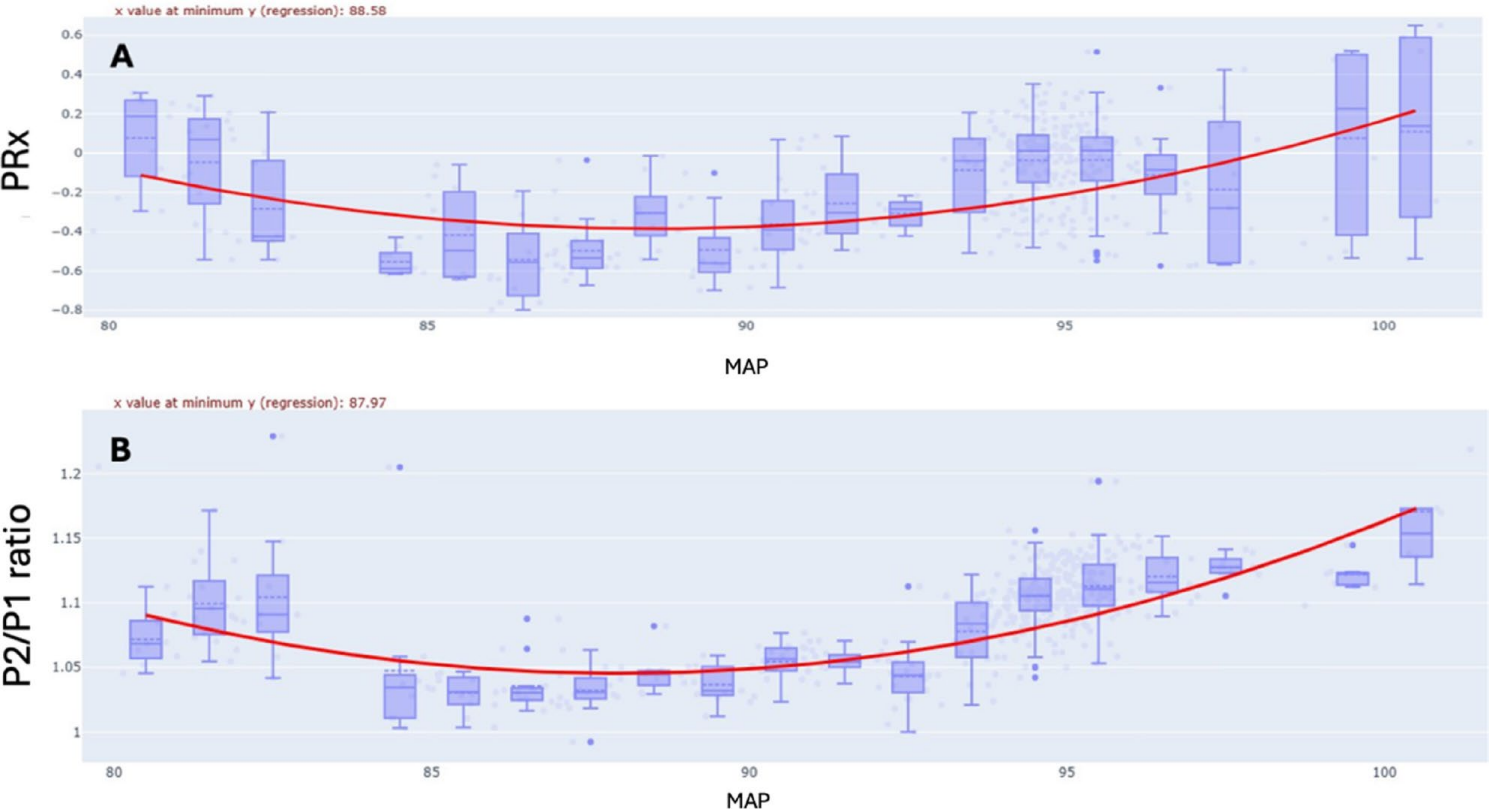
A single patient plot of optimal mean arterial pressure (MAP) assessment using PRx and P2/P1ratio. The upper panel (**A**) illustrates the U-shaped curve obtained from polynomial regression analysis of PRx (correlation between ICP and ABP), with the optimal MAP value (88.58 mmHg) indicated by the nadir of the red curve. The lower panel (**B**) presents the same analysis utilizing the P2/P1 ratio, demonstrating an optimal MAP value of 87.97 mmHg

**Fig. 3:**
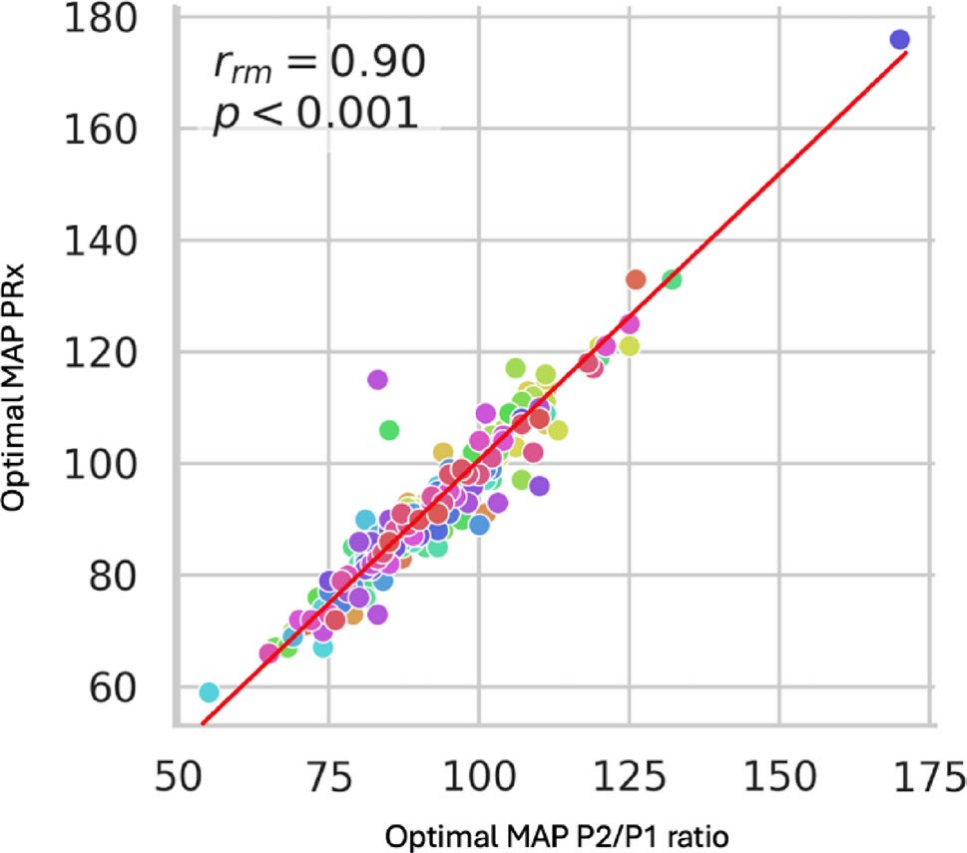
Relationship between the optimal mean arterial pressure (MAP) determined from the P2/P1 ratio and that obtained from the correlation between intracranial pressure and MAP (PRx). Each point represents an individual monitoring session. The red line represents the linear regression fit (y = 0.891x + 11.218)

**Fig. 4:**
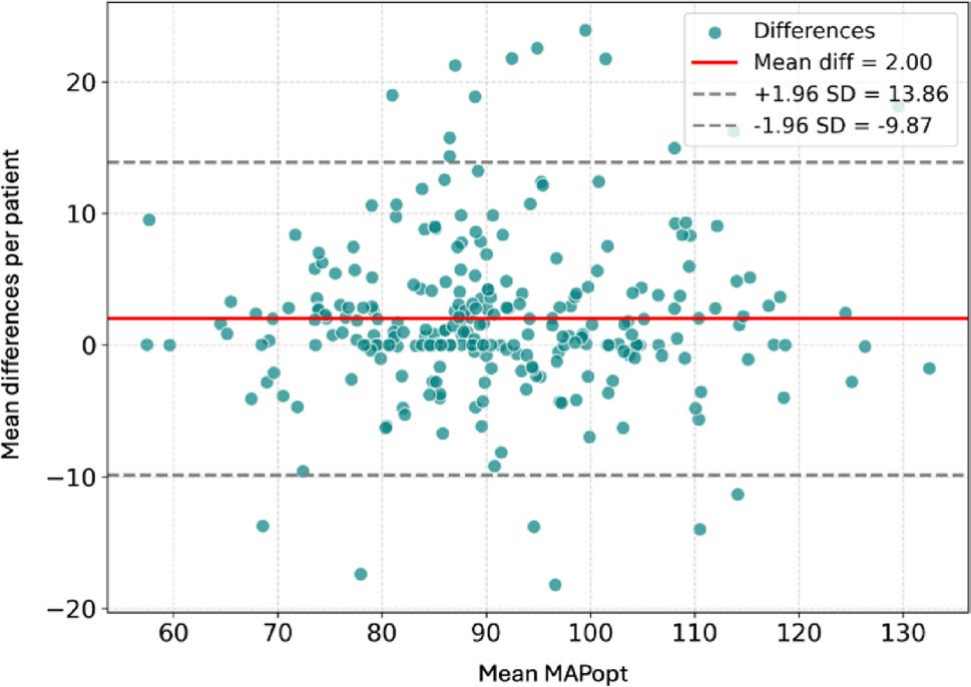
Bland–Altman plot illustrating the agreement between optimal mean arterial pressure (MAP) values determined by the correlation between intracranial pressure (ICP) and MAP, and those obtained from the P2/P1 ratio. The red line indicates the mean difference, while the dotted lines represent the 95% limits of agreement (± 1.96 SD)

**Fig. 5:**
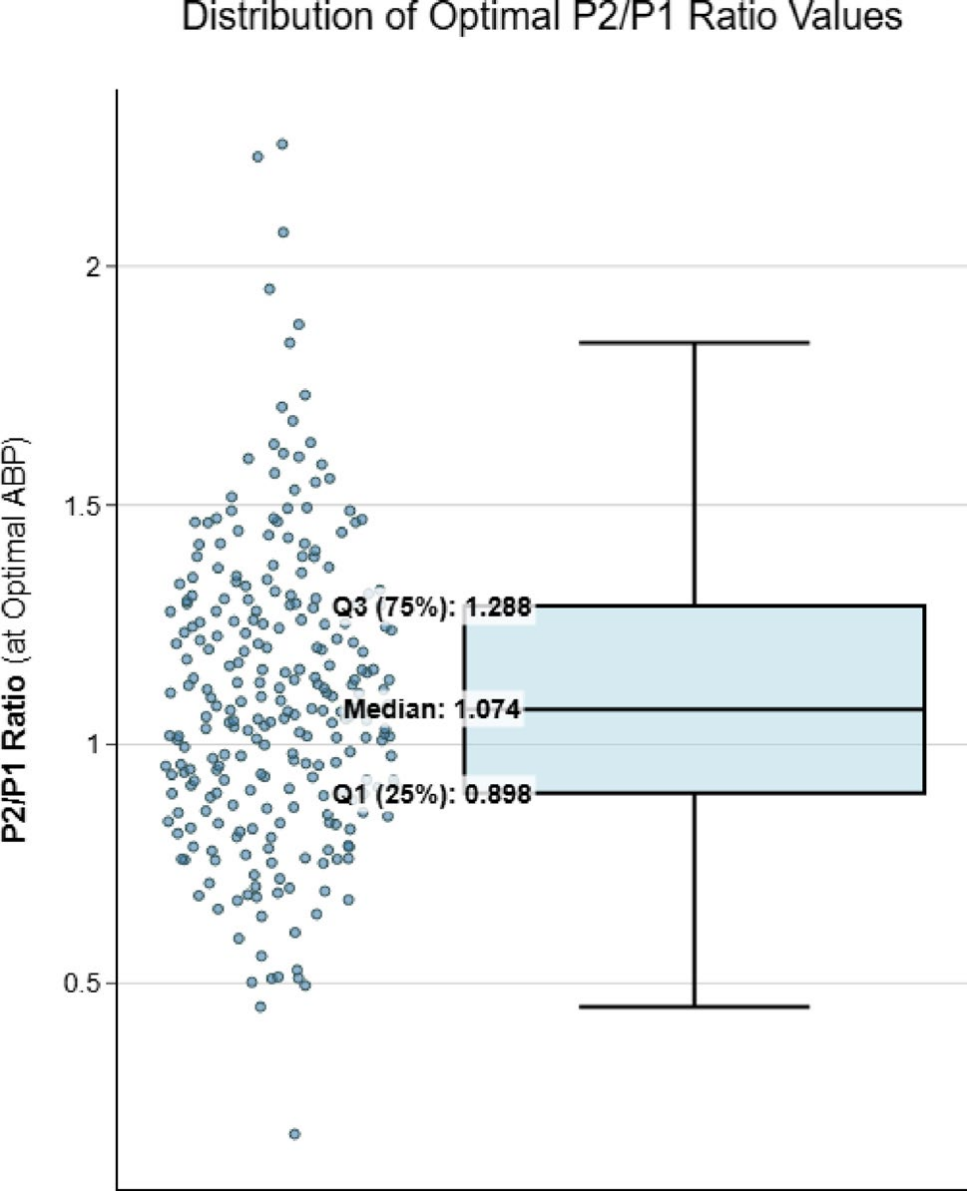
The median P2/P1 ratio value within the optimal MAP range was 1.074; the interquartile range (IQR) spanned from 0.8979 to 1.2877; and the mean ± standard deviation was 1.0979 ± 0.3057

An example from a single patient of optimal MAP identification by means of PRx and P2/P1 ratio is shown in Fig. 2. Analysis of optimal MAP for the entire sample demonstrated a strong linear relationship (rmcorr = 0.9, *p* < 0.0001), indicating that the arterial pressure corresponding to the ideal P2/P1 ratio range closely aligns with the pressure at which PRx also falls within its optimal range (Fig. 3). This finding supports the physiological coherence between noninvasive (B4C) and invasive (ICP) markers in defining the optimal MAP. Regarding agreement analysis (Bland-Altman), the mean difference between methods was + 2.00 mmHg. The 95% limits of agreement ranged from − 9.87 mmHg to + 13.86 mmHg, with 80% of cases demonstrating differences within 6 mmHg. The plot demonstrates good agreement without systematic bias across the pressure range, supporting the interchangeability of invasive (ICP-based) and noninvasive (B4C-based) estimates of optimal arterial pressure (Fig. 4). The study found that optimal MAP aligns with a P2/P1 ratio between 0.89 and 1.28, with a median value of 1.07 (Fig. 5).

## Discussion

These present results indicate the noninvasive P2/P1 ratio, a marker of intracranial compliance, can be used as a parameter to infer optimal systemic ABP, enabling personalized, bedside-guided therapeutic management. Optimizing CPP and MAP is a cornerstone in the management of patients with acute brain injury and long lasting surgeries [28], aiming to ensure adequate (CBF and minimize secondary insults [29–31]. However, determining the optimal MAP or CPPopt is complex due to inter-individual variability and the dynamic nature of CA. Current neurocritical care guidelines often recommend fixed CPP thresholds, typically ranging from 60 to 70 mmHg, but growing evidence suggests these generalized targets may not be optimal for all patients, as the ideal CPP can vary significantly depending on the dynamic state of their CA [4, 32]. The concept of “optimal CPP” has emerged, defined as the CPP (or MAP) range where CA is most effective, thereby potentially maximizing cerebral oxygenation and minimizing secondary injury [33]. Identifying this patient-specific optimal MAP is crucial for truly individualized neurocritical care [34, 35].

Our study aimed to validate a noninvasive approach for identifying MAPopt, utilizing the P2/P1 ratio derived from noninvasive intracranial pressure waveforms acquired by the B4C sensor, against the established gold-standard invasive method, the PRx. Our findings demonstrate a strong linear correlation between the noninvasive B4C-based optimal MAP and the invasive PRx-based optimal MAP. Furthermore, Bland-Altman analysis revealed good agreement between the two methods. This indicates minimal systematic bias across the pressure range, supporting the potential interchangeability of invasive (ICP-based) and noninvasive (B4C-based) estimates of optimal arterial pressure. These compelling results highlight the clinical viability of noninvasive optimal MAP monitoring, offering a significant step forward in advanced neuromonitoring in critical care.

Individualized CPP management is supported by recent literature [36]. Studies underscore the ongoing debate and research into which ICP-derived indices are most effective for predicting outcomes in specific conditions like moderate/severe TBI [37]. Likewise, individualized CPP targets have shown a positive impact on preventing delayed cerebral infarction and improving functional outcomes in spontaneous SAH [37]. These studies collectively emphasize the critical need for accurate and patient-specific CPPopt determination, providing a robust backdrop for the clinical utility of our findings.

The PRx has long been recognized as the most reliable real-time monitoring tool for assessing the integrity of CA and identifying optimal MAP [38, 39]. PRx is calculated as a moving correlation coefficient between mean ICP values and ABP [40]. Physiologically, when CA is intact, an increase in ABP leads to vasoconstriction, preventing a significant rise in ICP, resulting in a negative or near-zero PRx. Conversely, when CA is impaired, changes in ABP are passively transmitted to the cerebral vasculature, causing concomitant changes in ICP, leading to a positive PRx [41]. While highly effective, the primary drawback of PRx is its dependence on invasive ICP monitoring (typically requiring an intraparenchymal or ventricular catheter) and continuous arterial pressure monitoring. This invasive requirement inherently limits its applicability to critically ill patients, primarily in settings such as severe TBI, thereby restricting wider adoption in various neurocritical care scenarios or for patients who do not meet criteria for invasive ICP monitoring.

Our study introduces the noninvasive P2/P1 ratio as a compelling alternative. The P2/P1 ratio, derived from the morphology of the ICP pulse amplitude, reflects changes in intracranial dynamics and compliance [42]. This ratio has shown a very high negative predictive value in several studies [20, 22, 23]. ICP pulse amplitudes respond to changes in ABP or ICP, and their correlation with ABP can indicate specific ABP levels that lead to significant variations, often displaying a U-shaped curve with an optimal ABP nadir. The B4C system, a highly sensitive cranial deformation sensor, noninvasively detects tiny cranial expansions caused by changes in intracranial pressure with each heartbeat, and previous studies have consistently shown its waveforms correlate with invasive ICP measurements.

B4C pulse amplitudes have been shown to assess the pulse-amplitude index (PAx) [11]. PAx is a surrogate index for the PRx in the evaluation of ABP reactivity [33]. The strong linear relationship observed between the MAPopt derived from the noninvasive P2/P1 ratio and the invasive PRx is not merely a statistical correlation; it underscores a profound physiological coherence between these two distinct yet related measures. Both indices, when stratified by MAP, exhibit a U-shaped curve, with their respective nadirs representing the point of optimal CA [43]. This congruence strongly suggests that the noninvasive P2/P1 ratio, via the B4C sensor, is effectively capturing the same underlying physiological state of cerebral autoregulatory integrity as the invasive PRx.

The potential clinical implications of a noninvasive method for determining optimal MAP would be substantial. Foremost, it may significantly expand the scope of CA assessment beyond the confines of invasive ICP monitoring. Like dynamic bedside CA assessments with transcranial Doppler (TCD), intentionally changing MAP quickly reveals if CA is impaired and helps find each patient’s optimal MAP [44]. An advantage of this method, to be examined in future studies, is that while TCD needs dedicated software to identify the optimal MAP range, B4C can offer real-time results without extra analytics tools. If future research confirms these findings, this approach may help patients who cannot have invasive ICP monitoring, those at different stages of recovery, or even individuals in pre-hospital settings. Such expanded access to real-time CA status can lead to more personalized and precise hemodynamic management, potentially avoiding both cerebral hypoperfusion and hyperemia. The ability to titrate blood pressure therapies to a patient’s individual optimal MAP can lead to more effective preservation of brain tissue and improved neurological outcomes.

The implementation of a noninvasive tool may mitigate risks inherent to invasive procedures, such as infection or hemorrhage, while also contributing to the reduction of healthcare expenditures [45]. The call for more reliable and stable CPPopt algorithms [5] resonates deeply with our findings. The B4C system’s noninvasive nature, coupled with its demonstrated correlation to the gold standard, positions it as a valuable contributor to developing more robust autoregulation-oriented management strategies for ABI patients.

Building upon this foundational work, future research should focus on several key areas. Prospective, randomized controlled trials comparing optimal MAP-guided therapy using the B4C system against conventional or invasive PRx-guided approaches are essential to evaluate the impact on long-term neurological and functional outcomes. Investigating the applicability of the B4C sensor in other patient populations, such as those with less severe TBI, hydrocephalus, or in settings requiring outpatient or perioperative monitoring, could further broaden its clinical utility. The development of real-time feedback algorithms that integrate B4C data with other physiological parameters could further refine hemodynamic management strategies. Finally, exploring the noninvasive P2/P1 ratio as a potential biomarker for other neurological conditions, beyond optimal MAP identification, may uncover novel diagnostic and prognostic applications.

Although the findings are encouraging, this study is subject to several limitations. These include its retrospective design, a relatively limited sample size, and heterogeneity within the cohort, all of which may constrain the generalizability of the results to broader acute brain injury populations. The intermittent nature of data collection, involving brief recording sessions, may not fully capture the continuous and dynamic fluctuations of MAPopt over longer periods. Furthermore, the absence of intentional ABP manipulation means the full spectrum of CA responses was not extensively explored under varied physiological conditions. Critically, while the study establishes a strong methodological correlation, it does not directly assess the impact of noninvasive optimal MAP-guided therapy on patient-specific clinical outcomes. There are no studies to assess the possibility of significant differences for B4C parameters between the head sides to date. Finally, the exclusion of patients with conditions like decompressive craniectomy further limits the applicability of these findings to a broader neurocritical care population.

## Conclusions

Our study provides evidence that the P2/P1 ratio derived from noninvasive ICP waveforms, measured by the B4C sensor, is a reliable and accurate method for determining the optimal mean arterial pressure in patients with acute brain injury. The ability to monitor cerebral autoregulation more accessibly will open new avenues for individualized blood pressure management, with the potential to improve clinical outcomes and transform neurocritical care practice.

## Supplementary Information

Supplementary Material 1.

## Data Availability

Data are available from the corresponding author upon reasonable request.
