# Supplementary material for: A noninvasive method for assessing optimal cerebral perfusion pressure
Source: Crit Care. 2026 Mar 11;30:190. doi: 10.1186/s13054-026-05926-w (PMC13094200; doi:10.1186/s13054-026-05926-w)
Supplement: Supplementary file 1 — Supplementary Material 1. [file 13054_2026_5926_MOESM1_ESM.docx]

**A noninvasive method for assessing optimal cerebral perfusion pressure**

*Corresponding author:

Sérgio Brasil, MD, PhD

Division of Neurosurgery - University of São Paulo

sbrasil@alumni.usp.br

**Supplemental table.** STARD criteria for the study structure.

| **Section & Topic** | **No** | **Item** | **page #** |
| --- | --- | --- | --- |
| **TITLE** |  |  |  |
|  | **1** | Identification as a study of diagnostic accuracy using at least one measure of accuracy (such as association, correlation, sensitivity, specificity, predictive values, or AUC) | 3 |
| **ABSTRACT** |  |  |  |
|  | **2** | Structured summary of study design, methods, results, and conclusions (for specific guidance, see STARD for Abstracts) | 2 |
| **INTRO** |  |  |  |
|  | **3** | Scientific and clinical background, including the intended use and clinical role  of the index test | 3 |
|  | **4** | Study objectives and hypotheses | 4 |
| **METHODS** |  |  |  |
| *Study design* | **5** | Whether data collection was planned before the index test and reference  standard were performed (prospective study) or after (retrospective study) | 4 |
| *Participants* | **6** | Eligibility criteria | 4-5 |
|  | **7** | On what basis potentially eligible participants were identified  (such as symptoms, results from previous tests, inclusion in registry) | 4-5 |
|  | **8** | Where and when potentially eligible participants were identified (setting,  location and dates) | 5 |
|  | **9** | Whether participants formed a consecutive, random or convenience series | 5 |
| *Test methods* | **10a** | Index test, in sufficient detail to allow replication | 6 |
|  | **10b** | Reference standard, in sufficient detail to allow replication | 5 |
|  | **11** | Rationale for choosing the reference standard (if alternatives exist) | 5 |
|  | **12a** | Definition of and rationale for test positivity cut-offs or result categories  of the index test, distinguishing pre-specified from exploratory | 7 |
|  | **12b** | Definition of and rationale for test positivity cut-offs or result categories  of the reference standard, distinguishing pre-specified from exploratory | 7 |
|  | **13a** | Whether clinical information and reference standard results were available   to the performers/readers of the index test | 7 |
|  | **13b** | Whether clinical information and index test results were available to the  assessors of the reference standard | 7 |
